# Broad-range polymerase chain reaction and sequencing for the diagnosis of infectious diseases

**DOI:** 10.1128/spectrum.02505-24

**Published:** 2025-03-05

**Authors:** Nicole E. Putnam, Drew W. Charles, James B. Doub, J. Kristie Johnson

**Affiliations:** 1 Department of Pathology, University of Maryland School of Medicine, Baltimore, Maryland, USA; 2 Clinical Microbiology Laboratory, Laboratories of Pathology, University of Maryland Medical Center, Baltimore, Maryland, USA; 3 Division of Clinical Care and Research, Institute of Human Virology, University of Maryland School of Medicine, Baltimore, Maryland, USA; 4 Division of Infectious Disease, Medical University of South Carolina, Charleston, South Carolina, USA; bioMerieux Inc, Denver, Colorado, USA

**Keywords:** molecular diagnostics, broad range PCR, universal PCR, 16S rDNA, infectious disease diagnostics

## Abstract

Broad-range polymerase chain reaction (BR-PCR) identifies molecular signatures of microorganisms directly from clinical specimens without requiring microbial growth in culture. BR-PCR may be a powerful tool to reveal microbial causes of infectious diseases, but the impact on diagnosis and clinical management has yet to be fully defined. Consequently, the aims here were to investigate how bacterial, fungal, and mycobacterial (AFB) BR-PCR perform compared to microbiology culture methods in detecting microorganisms and to assess clinical utility, defined as the ability of the results to change antimicrobial therapy or treatment duration. Between 2018 and 2021, 348 unique specimens were sent from 327 patients seen within the University of Maryland Medical System (UMMS). Patient charts were reviewed retrospectively. Organisms identified by BR-PCR were compared to bacterial (*n* = 302), fungal (*n* = 137), and AFB (*n* = 111) cultures to determine concordance and were evaluated to determine if they led to a change in clinical management. Agreement in organism(s) reported by BR-PCR and culture was considered concordant for calculating performance data. Sensitivity of BR-PCR compared to concordant culture results was 30.9% for bacteria (25/81; 95% CI: 21.8–41.6%), 18.8% for fungi (3/16; 95% CI: 5.8–43.8%), and 33.3% for AFB (1/3; 95% CI: 5.6–79.8%) detection. The bacterial negative percent agreement of 80.1% (165/206) may reflect antibiotic pretreatment or detection of fastidious organisms. Despite longer turnaround times, BR-PCR results changed clinical care in 6% of cases. Based on these findings herein, the clinical use of BR-PCR would be best utilized when fastidious organisms are suspected, or specimens remain culture negative, but should not replace routine culture methods at this time.

**IMPORTANCE:**

Determining infectious etiology can be challenging in patients with chronic presentation and in those receiving empiric therapy. In addition to the standard of care (microbiology cultures), providers can order a broad-range polymerase chain reaction and sequencing (BR-PCR) test to identify microorganisms directly from clinical specimens and independently from culture. While studies have been done from individual hospitals or systems, there is a lack of broadly applicable clinical evidence detailing clinical scenarios in which BR-PCR should be utilized. This study adds to the growing body of literature surrounding BR-PCR clinical usage, examining assay performance and clinical utility of BR-PCR test results. Although BR-PCR and culture had low concordance among organisms identified, it was shown to complement the standard of care for uncommonly isolated and fastidious organisms. Overall, BR-PCR results changed clinical management in 6% of cases, which is similar to other studies that include a broad representation of specimen types.

## INTRODUCTION

Determining the etiology of infections is paramount to direct antimicrobial therapies, which not only result in cure but also allow for antibiotic coverage to be narrowed, thereby eliminating side effects from broad-spectrum antibiotics (1, 2). The gold standard in diagnosing most infections is microbial culture (1, 2). However, cultures are often obscured by empiric antibiotic usage, and chronic infections are often associated with microbes in sessile states, limiting the ability to recover microbes with standard culture techniques (3). As a result, some infectious disease guidelines recommend treating culture-negative infections (4, 5). Consequently, there is a drastic need to develop diagnostics that elucidate microbial causes of infection in conjunction with microbial cultures.

One such diagnostic technique is broad-range polymerase chain reaction and sequencing (BR-PCR) (6, 7). This diagnostic test identifies molecular signatures of microorganisms directly from clinical specimens without requiring microbial growth in culture. BR-PCR utilizes molecular methods to amplify common gene targets with hypervariable regions that have organism- and species-specific differences. Sequencing of the amplified gene products and comparison with validated databases allow for microorganisms present in the sample to be identified. Bacteria are targeted using the 16S ribosomal DNA sequence (7), and fungi are identified with the polymorphic internal transcribed spacer (ITS) regions ITS1 and ITS2 within ribosomal DNA and/or the 28S ribosomal DNA sequence (8–10). Advancements in technology now allow for multiple organism templates to be detected and identified in parallel using next-generation sequencing (NGS). Identification of AFB, including nontuberculous *Mycobacteria* (NTM), *M. tuberculosis,* and *M. avium* complexes, can use various DNA targets including 16S ribosomal DNA, *rpoB*, and *hsp65* (11).

BR-PCR is a promising diagnostic but, at present time, is widely ordered without substantial data to inform the optimal scenarios for utilization. Updated guidelines for microbiology test utilization from the Infectious Diseases Society of America (IDSA) and American Society of Microbiology (ASM) suggest that when an uncommon infectious diagnosis is suspected, consultation with the laboratory director should occur to evaluate the need for specialized techniques (12). 16S BR-PCR is discussed for body sites, including endovascular, central nervous system, bone, and joint infections, but the guidelines highlight that molecular testing from these samples is not recommended as a first-line diagnostic test and should be performed in conjunction with Gram stain or pathology reports (12). BR-PCR can be a powerful tool to reveal microbial causes of infections (13–15), but the sensitivity, specificity, and impact of BR-PCR results on clinical management are still being defined (13, 16–20). Therefore, the aims of this study were to investigate how bacterial, fungal, and mycobacterial (acid-fast bacilli, AFB) BR-PCR performed compared to standard-of-care microbiology culture methods and with respect to changes in clinical management.

## MATERIALS AND METHODS

### Study design

We performed a retrospective cross-sectional study evaluating the effect of BR-PCR results on the diagnosis and management of University of Maryland Medical System (UMMS) patients between 2018 and 2021. A total of 420 specimens were sent for BR-PCR to the University of Washington during this 4-year period, with 67 specimens excluded that represented replicate samples. If distinct specimens from the same collection were sent with individual BR-PCR orders, orders and results were condensed so that replicate specimens, characteristics, and findings were not overrepresented. As with culture, any positive result was meaningful.

### Population and specimen characteristics

Electronic medical records (EMR) were reviewed for demographics and comorbidities (Table 1). Specimens from BR-PCR test orders were characterized by body site and specimen type (Table 2). Various body sites represented include bone and joint, central nervous system (CNS), endovascular (endocarditis, vascular grafts), intra-abdominal (liver, peritoneal), intrathoracic (pulmonary, mediastinal), ophthalmic, or soft tissue. Specimen types were characterized as fresh frozen tissue, formalin-fixed paraffin-embedded (FFPE) tissue, or body fluid, along with data regarding the length of antimicrobial treatment the patient received prior to specimen collection. These parameters were then evaluated to determine patient or specimen characteristics with a higher likelihood of having a positive BR-PCR result. TAT was calculated as the time elapsed between specimen collection and the posting of results to the EMR.

**TABLE 1:** Study population demographics

Number of subjects (*n*)		327
Age, years (mean ± SD)		53 ± 16
Gender	*n*	%
Male	190	58.10%
Female	137	41.90%
Race	*n*	%
White	169	51.70%
Black	128	39.10%
Asian	12	3.70%
Hispanic	12	3.70%
Other	6	1.80%
Immunodeficient	*n*	%
Total	111	33.90%
Autoimmune	14	4.30%
Cancer	24	7.30%
HSCT	3	0.90%
Immunosuppressive therapy	7	2.10%
Other	7	2.10%
Primary immunodeficiency	6	1.90%
SOT	50	15.30%
Diabetic	68	20.80%

**TABLE 2:** Specimen characteristics

Specimens (*n*)	348	
Antimicrobial therapy prior to specimen collection	*n*	%
No	100	28.70%
Yes	248	71.30%
If yes, duration of therapy (*n* = 252)	*n*	%
1–2 days	56	22.60%
3–7 days	91	36.70%
>1–4 weeks	71	28.60%
>4 weeks	30	12.10%
Body site	*n*	%
Bone and joint	120	34.50%
CNS	60	17.20%
Endovascular	52	14.90%
Intra-abdominal	30	8.60%
Intrathoracic	48	13.80%
Ophthalmic	2	0.60%
Soft tissue	36	10.30%
Specimen type	*n*	%
Fresh tissue	187	53.70%
FFPE	55	15.80%
Body fluid	106	30.50%

**Fig 1:**
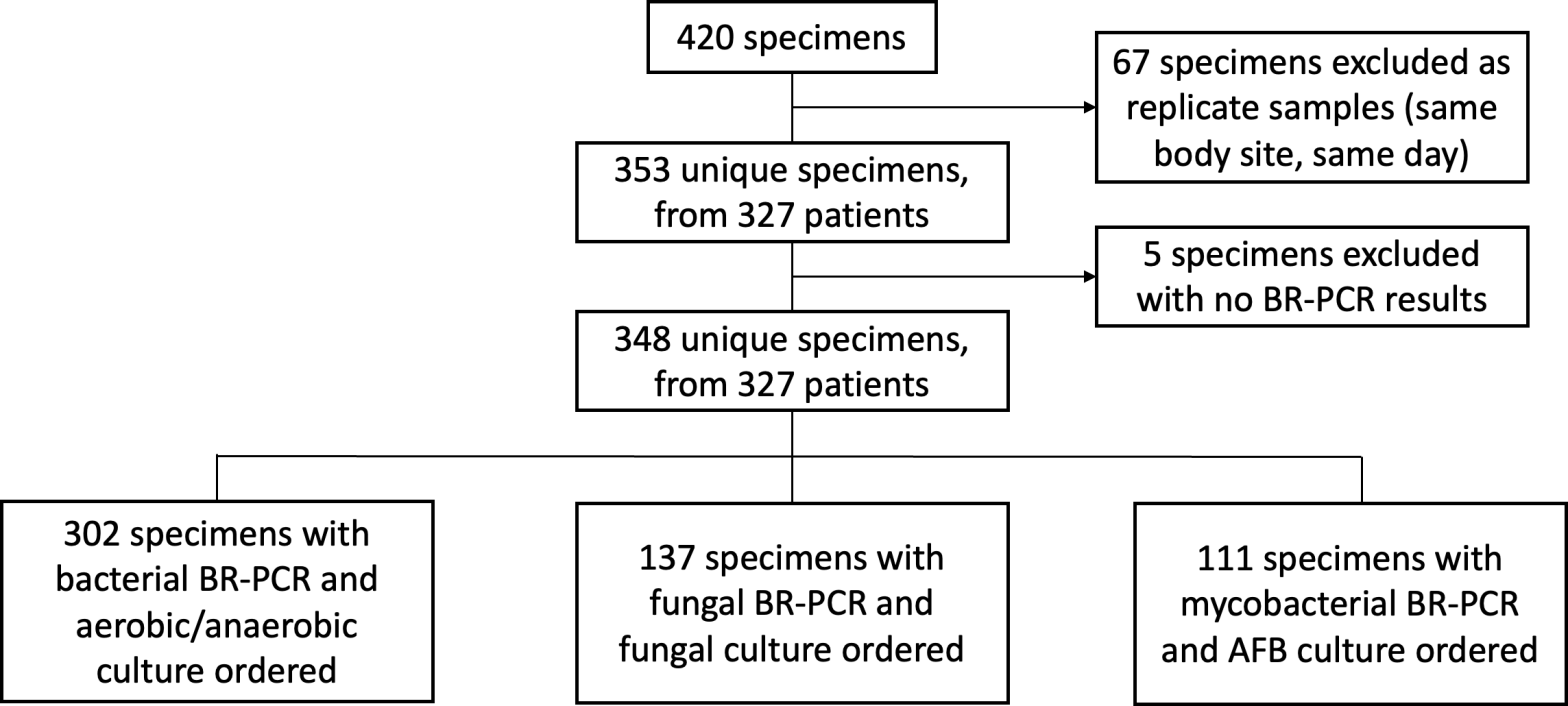
Retrospective chart review data and samples included in each study comparison. All specimens sent for BR-PCR at UMMC between 2018 and 2021. Replicate samples (*n* = 67) were collapsed so all BR-PCR results were taken into account on duplicate specimens. Data were not assessed for specimens (*n* = 5), where no BR-PCR results were found uploaded to the electronic health record. Of the 348 unique specimens sent from 327 patients, descriptive comparisons and statistics were conducted only if BR-PCR and culture were ordered and performed for each organism type: bacteria, fungi, and mycobacteria.

Results from microbiology, pathology, and BR-PCR tests from 348 unique specimens collected from 327 patients were evaluated in this study. Study design and exclusions are described in Fig. 1. We assessed BR-PCR positivity by organism type and body site (Table 3), and a binary comparison of BR-PCR and culture test positivity was done for bacteria (*n* = 302), fungi (*n* = 137), and AFB (*n* = 111) (Table 4A through C). BR-PCR and culture reports from each specimen were subsequently compared for concordance of all organisms identified. Agreement in organism(s) reported was considered concordant for the purpose of calculating the performance data (Table 4D through F). If result comparisons were obscured by reporting differences, but these could likely represent the same organism(s), they were considered concordant. Examples of concordant results with reporting differences are “Anaerobic Gram-positive rod” reported by culture and “*Clostridium disporicum*” reported by BR-PCR (Table S1). Samples with organism(s) reported by one methodology only were considered discordant. A change in clinical management in response to BR-PCR results was defined as a documented change in antimicrobial therapy, as interpreted by provider notes and availability of BR-PCR results. A consensus among authors reviewing all suspect cases was reached. A change in clinical management of the patient’s condition was subcategorized as (1) a change in antimicrobial agent or duration of therapy or (2) the termination of antimicrobial therapy.

**TABLE 3:** Percent positivity of BR-PCR orders by body site and organism type

Body site	Bacterial BR-PCR	Fungal BR-PCR	AFB BR-PCR
	Positive/total (%)	Positive/total (%)	Positive/total (%)
Bone and joint	33/118 (28.0%)	4/74 (5.4%)	0/67 (0%)
Central nervous system	14/59 (23.7%)	1/38 (2.6%)	0/33 (0%)
Endovascular	22/52 (42.3%)	2/35 (5.7%)	0/29 (0%)
Intra-abdominal	12/26 (46.2%)	1/17 (5.9%)	0/17 (0%)
Intrathoracic	16/45 (35.6%)	2/34 (5.9%)	2/33 (6.1%)
Ophthalmic	0/2 (0%)	0/1 (0%)	0/0
Soft tissue	13/35 (37.1%)	7/33 (21.2%)	2/27 (7.4%)
All	110/337 (32.6%)	17/232 (7.3%)	4/206 (1.9%)

**TABLE 4:** Culture versus BR-PCR positivity and descriptive statistics using concordant results

	A. Bacterial detection				B. Fungal detection				C. Mycobacterial detection			
		Bacterial culture				Fungal culture				AFB culture		
		Pos	Neg	Total		Pos	Neg	Total		Pos	Neg	Total
BR-PCR	Pos	40	56	96	Pos	5	4	9	Pos	1	0	1
	Neg	41	165	206	Neg	11	117	128	Neg	2	108	110
	Total	81	221	302	Total	16	121	137	Total	3	108	111
	D. Bacterial				E. Fungal				F. Mycobacterial			
NPA	165/206 (80.1%,				117/128 (91.4%,				108/110 (98.1%,			
	95% CI: 74.1–85.0%)				95% CI: 85.1–95.3%)				95% CI: 93.2–99.9%)			
PPA	25/96 (26.0%,				3/9 (33.3%,				1/1 (100%,			
	95% CI: 18.3–35.7%)				95% CI: 11.7–64.9%)				95% CI: 16.8–100%)			
SENS	25/81 (30.9%,				3/16 (18.8%,				1/3 (33.3%,			
	95% CI: 21.8–41.6%)				95% CI: 5.8–43.8%)				95% CI: 5.6–79.8%)			
SPEC	165/221 (41.6%,				117/121 (96.7%,				108/108 (100%,			
	95% CI: 68.5–80.0%)				95% CI: 91.5–99.0%)				95% CI: 95.9–100%)			
Overall	190/302 (62.9%,				120/137 (87.6%,				109/111 (98.2%,			
Concordance	95% CI: 57.3–68.2%)				95% CI: 80.9–92.2%)				95% CI: 93.3–99.9%)			

### Statistics

Organisms that were positive for both BR-PCR and culture, but with discordant findings, were excluded from the descriptive statistics analysis. Assessment of BR-PCR performance for each organism category was then compared to the reference standard, culture, to include negative percent agreement (NPA), positive percent agreement (PPA), sensitivity, and specificity, with 95% confidence intervals calculated by modified-Wald method in GraphPad quick calcs. The overall concordance was calculated in relation to the total number of specimens with BR-PCR and equivalent culture ordered.

To determine the influence of factors on BR-PCR test positivity, a two-tailed Fisher’s exact test was used to compare the parameters including population demographics and specimen characteristics. Odds ratios were calculated to assess the frequency of various factors among BR-PCR-positive and -negative groups. The two-tailed Fisher’s exact test was used to determine comparability between culture and BR-PCR. Specimen type was compared by χ test for sample sizes >300 (bacterial BR-PCR) and by Fisher’s exact test with sample size <300 (fungal and AFB BR-PCR) using a 2 × 3 contingency table. TATs were compared between tests used for diagnosis of bacteria, fungi, and AFB using the Kruskal-Wallis nonparametric test and corrected with Dunn’s multiple comparisons. For all the tests, significance was set by ɑ = 0.05.

## RESULTS

### Study characteristics

This cohort of 327 patients was predominantly male (58.1%) and white (51.7%) (Table 1). Over one-third of the patients were immunocompromised (*n* = 111/327, 33.9%) and one-fifth were diabetic (*n* = 68/327, 20.8%) (Table 1). Most specimens (*n* = 248/348, 71.3%) were collected after antimicrobial therapy administration, ranging from 1 to 2 days to greater than 4 weeks in duration (Table 2). Bone and joints were the body sites most highly represented, with over one-third (*n* = 123, 34.9%) of all specimens (Table 2). Specimens were sent from various anatomic locations, including the CNS (*n* = 61, 17.2%), endovascular (*n* = 52, 14.7%), intra-abdominal (*n* = 30, 8.5%), intrathoracic (*n* = 49, 13.9%), ophthalmic (*n* = 2, 0.6%), and soft tissue (*n* = 36, 10.2%) (Table 2).

Sex, race, immunocompromised, and diabetic status can individually lead to a predisposition to certain infections (21–23), but these factors did not significantly influence BR-PCR test results in this cohort. Patients who had received antimicrobial treatment were 1.8 times more likely to have BR-PCR results positive for bacteria (75/210) than those without empiric treatment (21/92) (*P* = 0.03); however, stratified data by treatment duration did not show any significant differences. When comparing antimicrobial pretreatment between specimens with disparate test results, culture-negative BR-PCR-positive specimens were more likely to have been exposed to treatment prior to collection than culture-positive BR-PCR-negative specimens.

Bacteria were 5.5 times more likely to be detected by BR-PCR if Gram stain reported the presence of organisms (*P* < 0.00001) and 2.4 times more likely if inflammation was observed as the presence of neutrophils on Gram stain (*P* = 0.03) or noted in the pathology report (*P* = 0.005). Immunocompromised status, antimicrobial therapy prior to specimen collection, and signs of inflammation were not associated with an increased likelihood of detecting fungi or AFB by BR-PCR.

### Detection of microorganisms with BR-PCR

Across the 348 specimens, providers ordered a total of bacterial (*n* = 337), fungal (*n* = 232), and AFB (*n* = 206) BR-PCRs. Table 3 presents the percentage positivity of BR-PCR orders by body site. The overall bacterial BR-PCR had the highest positive percentages across body sites, with bacteria detected from intra-abdominal (12/26, 46.2%), endovascular (22/52, 42.3%), soft tissue (13/35, 37.1%), intrathoracic (16/45, 35.6%), bone and joint (33/118, 28.0%), and central nervous system (14/59, 23.7%) collections (Table 3). Soft tissue samples had the highest yield in detecting fungi (7/33, 21.2%) and AFB (2/27, 7.4%) by BR-PCR (Table 3).

**Fig 2:**
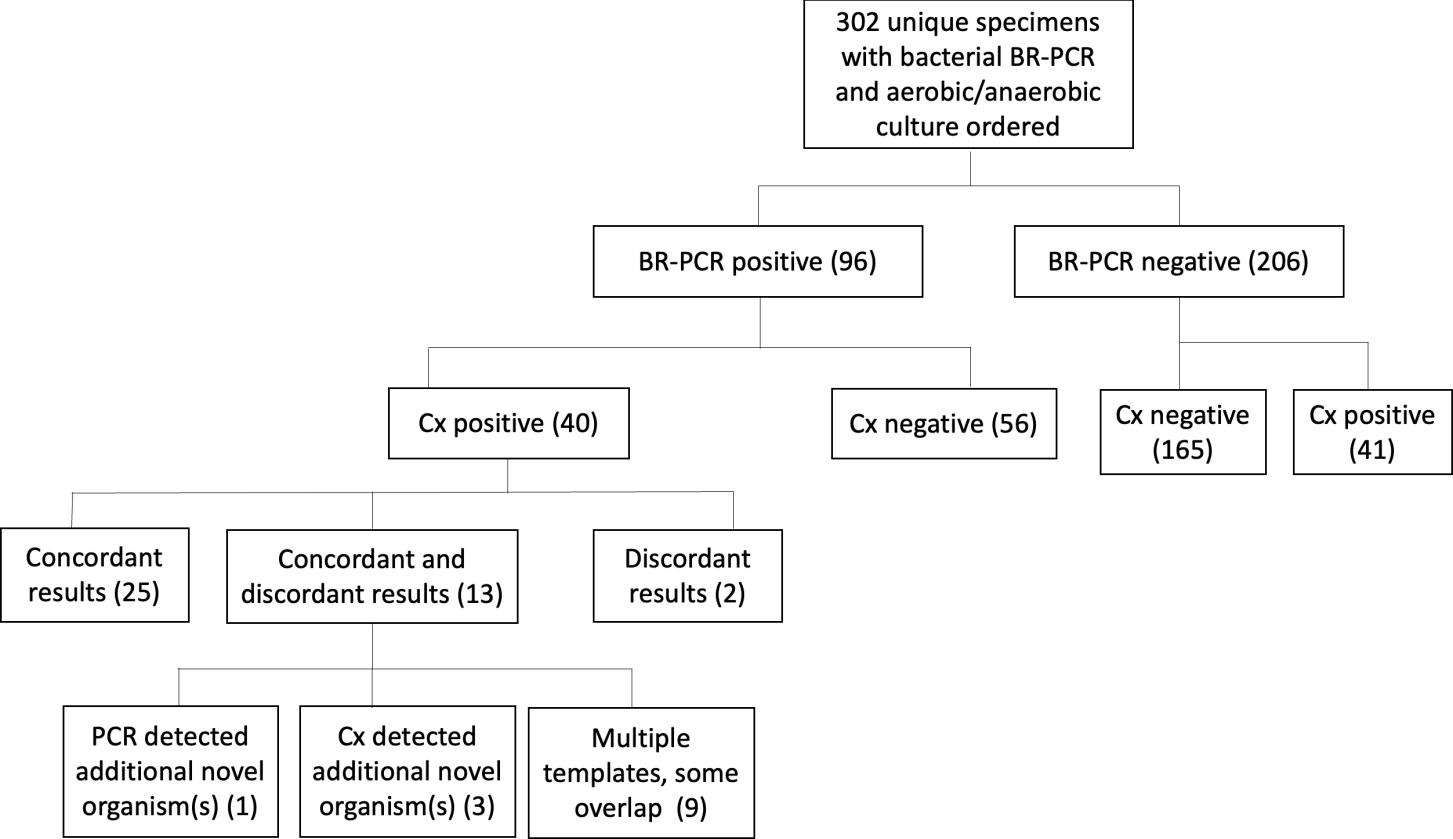
Bacterial BR-PCR and culture results. Bacterial BR-PCR positivity shows little correlation to culture positivity. Of BR-PCR-positive, culture (Cx)-positive specimens, results were compared for concordance: 25 specimens had concordant findings, two specimens had discordant findings, and 13 specimens had results that were more convoluted, with concordance for some organism(s) and discordance where BR-PCR (*n* = 1), Cx (*n* = 3), or both methodologies (*n* = 9) detected additional organism(s) missed by the other.

**Fig 3:**
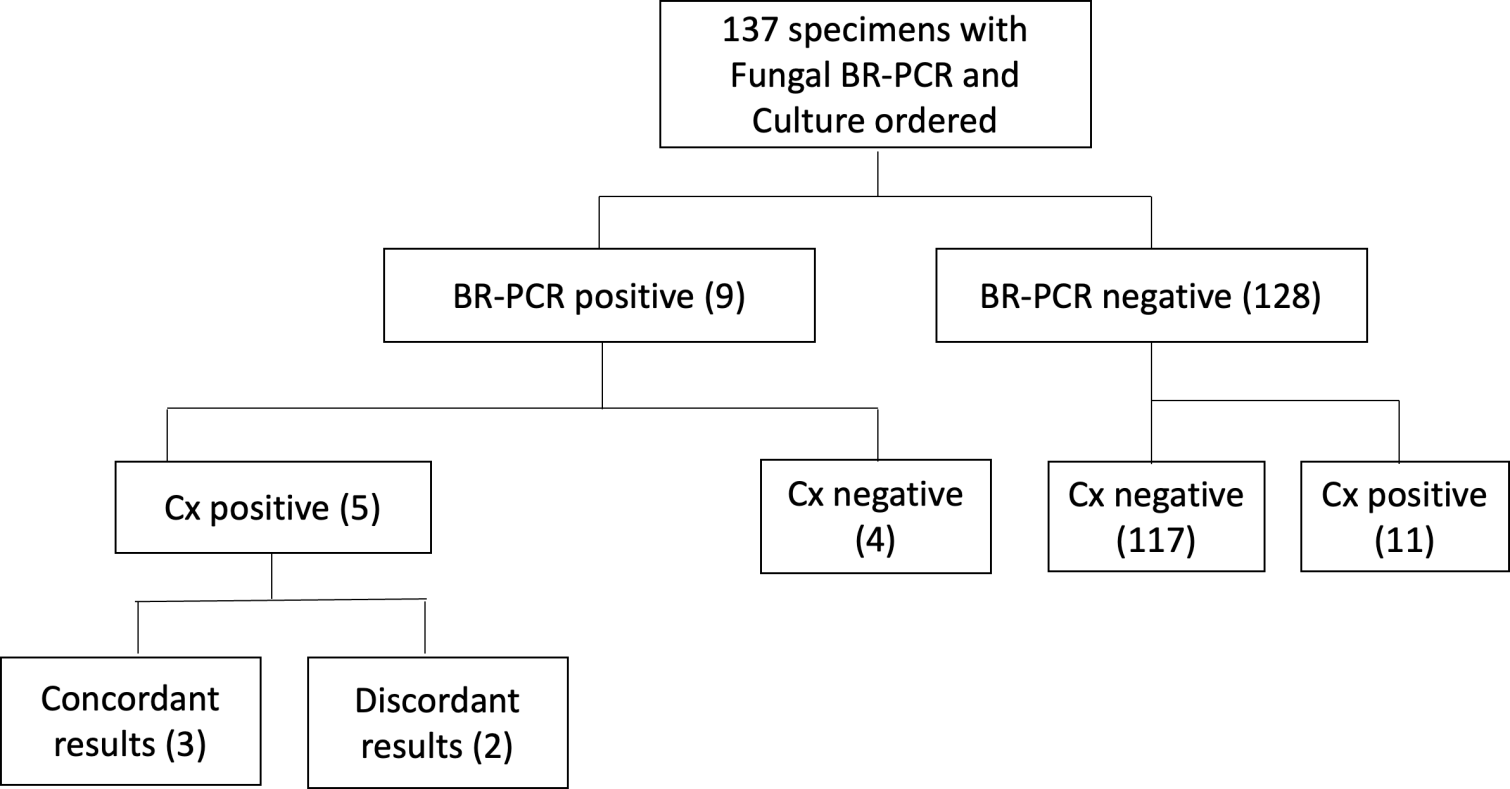
Fungal BR-PCR and culture results. Positivity can be viewed in relation to fungal BR-PCR and culture results. Of the BR-PCR positive, Cx positive specimens, results were compared for concordance. This revealed three concordant specimens, where PCR and Cx results detected the same fungal organism and two specimens with discordant organisms.

**Fig 4:**
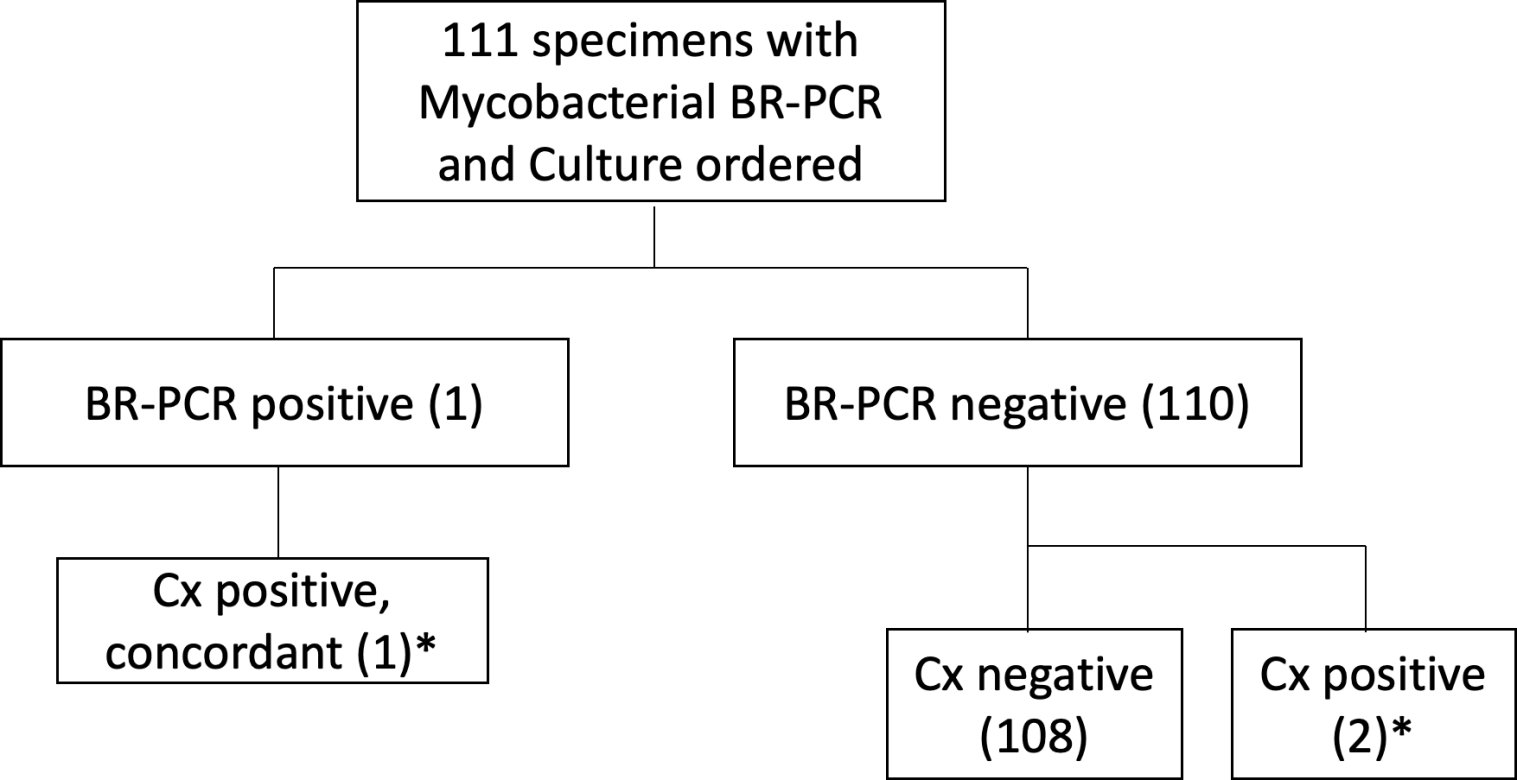
AFB BR-PCR and culture results. One specimen was positive for AFB BR-PCR and culture, detecting the same organism. As there was only one specimen with a positive AFB BR-PCR, there were no discrepancies in mycobacterial organisms detected. *All positive results from these 111 specimens detected *Mycobacterium tuberculosis* complex.

A binary comparison of positivity between bacterial BR-PCR and aerobic/anaerobic culture recognized 40 out of 302 specimens as positive by both test methods (Table 4A). Disparate abilities to detect bacteria occurred in 41 culture-positive specimens and 56 BR-PCR-positive specimens, where the other test method resulted negative (Fig. 2; Table 4A). Overall, few specimens were positive for fungi and AFB. Five out of the 137 specimens sent for fungal BR-PCR and culture were positive by both methods (Table 4B). Four BR-PCR-positive specimens and 11 culture-positive specimens showed contradictory fungal test results (Fig. 3; Table 4B). One out of 111 specimens sent for AFB testing was positive by both BR-PCR and culture, and two specimens recovered AFB in culture but not by AFB BR-PCR (Fig. 4; Table 4C). BR-PCR and culture were significantly different in their ability to detect bacteria (*P* = 0.0001), fungi (*P* = 0.0011), and AFB (*P* = 0.0270).

### Concordant microbe detection between BR-PCR and culture

Six specimens had multiple bacterial templates that could not be resolved by standard bacterial BR-PCR and/or NGS. One culture grew multiple organisms, and here, “multiple templates” were interpreted as a matching result, although there was no further resolution. BR-PCR reported up to ten microbes per specimen, compared to culture where specimens with more than three organisms per culture were reported as microbial flora, not including individual organism resolution. Twenty-five specimens identified the same bacteria in 302 specimens tested by bacterial BR-PCR and culture (Fig. 2). Concordance was complicated by polymicrobial specimens, where 13 specimens displayed some overlap in bacterial organism(s), where either bacterial BR-PCR (*n* = 1), bacterial culture (*n* = 3), or a combination of both (*n* = 9) had detected organisms not identified by the other methodology (Fig. 2). Bacterial identifications from BR-PCR- and culture-positive specimens and frequency of detection are listed in Table S1. Overall bacterial concordance was low at 62.9% (95% CI: 57.3–68.2%) (Table 4D), as many bacteria were detected solely by BR-PCR or culture (Tables S2 and S3). BR-PCR detected *Legionella* sp., *Mycoplasma hominis*, *Rhodococcus fascians*, *Ureaplasma* sp., and others not identified by infectious workup and routinely identified non-fastidious organisms, such as staphylococci, streptococci, likely environmental contaminants, and normal flora, depending on the body site.

Only three specimens had concordant fungal findings by BR-PCR and culture (Fig. 3; Table S4). It appears that filamentous molds were preferentially detected by BR-PCR in culture-negative specimens (Table S5). A higher number of specimens positive by fungal culture alone may suggest that culture is better able to recover fungi compared to BR-PCR, although the primary organisms detected by culture were *Candida* species (Table S6). Fungal BR-PCR detected mostly molds, some of which are particularly challenging to recover in culture*,* including *Malassezia* (*n* = 2), which requires long-chain fatty acids (e.g., olive oil overlay) in culture, and *Rhizopus* (*n* = 2), a delicate mold that is easily susceptible to destruction during tissue processing. *Rhizopus* was detected by pathology for both patients, with concern for fungal sinusitis and cutaneous disease. High fungal NPA (91.4%, 95% CI: 85.1–95.3%) and specificity (96.7%, 95% CI: 91.5–99.0%), driven in part by a large number of specimens negative for fungi, lead to a modest overall concordance of 87.6% (95% CI: 80.9–92.2%) (Table 3E).

Of the 111 specimens with paired tests (BR-PCR and culture) sent for diagnosis of AFB, *M. tuberculosis* complex was recovered in three AFB cultures but detected by AFB BR-PCR in just one of these specimens (Fig. 4). While overall concordance of AFB results was 98.2% (95% CI: 93.3–99.9%), sensitivity was just 33.3% (95% CI: 5.6–79.8%) (Table 3F).

### BR-PCR performance without reference standard culture

Many BR-PCR orders were placed without the corresponding culture: 35 specimens were sent for bacterial BR-PCR without aerobic/anaerobic culture, and 95 specimens each were sent for fungal and AFB BR-PCR with accompanying fungal and AFB cultures. Notably, 14 of the 35 specimens sent for bacterial BR-PCR only were positive, including six polymicrobial bacterial samples (Table S7). Fungal BR-PCR was positive in 8 out of 95 specimens without culture, which detected several filamentous molds and three *Candida* species (Table S7). Finally, mycobacteria were detected by AFB BR-PCR in three out of 95 specimens sent without accompanying AFB cultures (Table S7).

### Turnaround time

**Fig 5:**
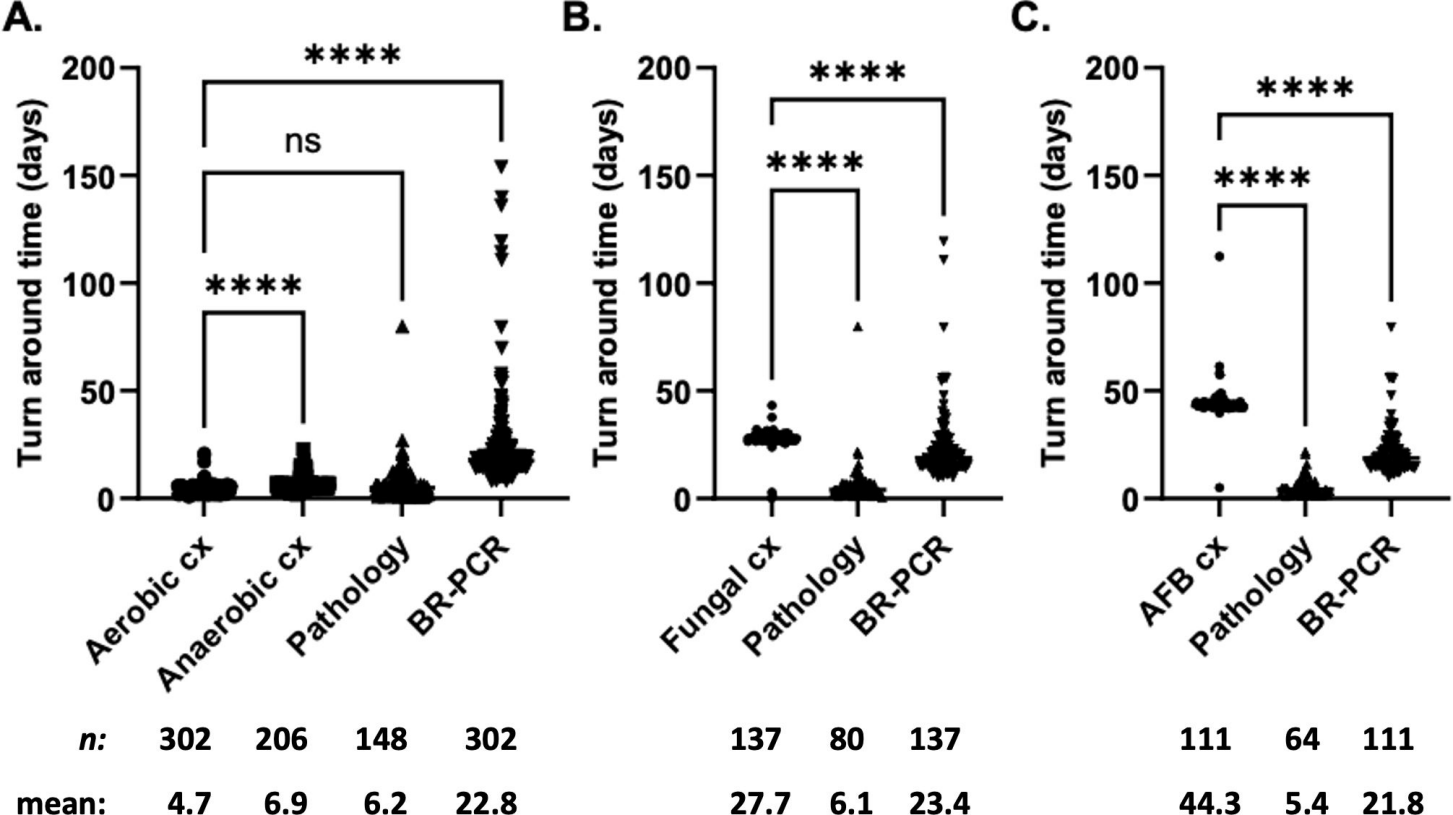
Result turnaround time (TAT). TAT was compared within each organism category (A, bacteria; B, fungi; C, mycobacteria) on specimens with orders for culture and corresponding BR-PCR using a Kruskal-Wallis non-parametric test corrected with Dunn's multiple comparisons. TAT was also considered from specimens in this subset with pathology orders. Below each test methodology, see the total number of samples (N) and the mean. ns, not significant, **** *P*<0.0001.

Aerobic culture results and pathology reports were available approximately 5 days after specimen collection, with anaerobic culture taking nearly 7 days (Fig. 5A). BR-PCR results for all organism groups were available to providers between 21 and 23 days, which was significantly longer than bacterial culture results (4.7 days) but sooner than fungal (27.7 days) and AFB (44.3 days) culture results (Fig. 5A through C).

### Clinical utility

BR-PCR results from 21 of 348 specimens (6%, 95% CI: 3.9–9.1%) contributed to a change in antimicrobial management with 16 positive (4.6%, 95% CI: 2.8–7.4%) and five negative (1.4%, 95% CI: 0.5-3.4%) BR-PCR results. Details of the case, results, and change in management are outlined in Table 5. Of the positive BR-PCR results leading to a change in clinical management of the patient, 25% (4/16) did not have appropriate microbiology orders to detect the microorganism(s) identified, despite their routine detection in culture (e.g., aerobic, anaerobic, fungal, AFB). Fastidious organisms, such as *Legionella* and *Mycoplasma*, which are not commonly isolated in standard culture,were identified by BR-PCR and led to a clinical change for patients (Table 5). Chart review revealed that many pathogens identified only by BR-PCR matched organisms recovered in culture from previous episodes of bacteremia, endocarditis, and hardware infections, although current specimen cultures remained negative (Table 5). Positive results in these scenarios provided evidence for providers to narrow or broaden therapy to encompass additional microorganisms. Negative BR-PCR results were one piece of information used to discontinue therapy, with other diagnostic test results, stable imaging, Gram stain/culture results, and clinical picture (Table 5).

**TABLE 5:** Utility of BR-PCR to change clinical management^*a*^^,^*^b^*

Age	Source	BR-PCR results	Culture result	Antimicrobial therapy change	Notes
65	CNS	*Acinetobacter johnsonii*	Negative	Vanc > Mero, Mino	s/*P* craniotomy for brain abscess I&D; later considered a contaminant
64	Bone, joint	*Aspergillus fumigatus* ^*c*^	N/A	Vori > Posa	Sternal wound post-surgery; polymicrobial
67	Bone, joint	*Cutibacterium acnes*	Negative	Vanc > Vanc, Amox-Clav	Chronic osteomyelitis
78	Endovascular	*Cutibacterium* species	Negative	Vanc > Erta, Linezolid	GPR on Gram stain; explantation of infected graft
59	Bone, joint	*Enterobacterales* family	Negative	Linezolid, Mero, Posa > Linezolid	New discitis/osteomyelitis
63	Soft tissue	*Fusarium penzigii*	Negative	Vanc > Vori	Total knee arthroplasty 2 years prior; persistent pain/swelling
26	Endovascular	*Haemophilus parainfluenzae*	Negative	Vanc, CTX > CTX	PVE; blood cultures with *S. sanguinis*, *H. parainfluenzae*, *Bacillus* sp.; *Bacillus* considered a contaminant
60	Intra-abdominal	*Legionella* species	negative	Dapto, Mero, Levo > Levo	Unclear significance, patient unresponsive to current therapy
47	Soft tissue	*Mycobacterium haemophilum* ^*c*^	N/A	> Azithro, Moxi, Linezolid	Skin biopsy of subcutaneous nodules; SOT patient
67	Bone, joint	*Mycoplasma hominis*	Negative	Amox-Clav > Doxy	Respiratory illness, retrosternal infection
76	CNS	*Rhodococcus fascians* ^*c*^	N/A	> Vanc, Mero, Azithro	Brain biopsy, altered mental status
69	Bone, joint	*Streptococcus mitis*, *Finegoldia* species, *Prevotella* species^*c*^	*S. mitis/oralis*	Vanc > Vanc, Amox-Clav	h/o prior PJI infections: *Pseudomonas aeruginosa*, CoNS
28	Endovascular	*Staphylococcus aureus*	Negative	CTX > Cefazolin	MSSA endocarditis, IVDU; stopped coverage for dog bite
63	Endovascular	*Staphylococcus epidermidis*	Negative	Mero, Vanc > Vanc	PVE, recurrent CoNS bacteremia; removed broad coverage for ostomy wound
80	Bone, joint	*Streptococcus dysgalactiae*	Negative	Dapto, CTX > CTX, Metronidazole	Poor dentition, hardware at site
53	Endovascular	*Streptococcus mitis* group	Negative	Ceftaroline > CTX	Incompletely treated bacteremia 1 month prior
31	Endovascular	Negative	*Corynebacterium acnes, Enterococcus faecalis*	Stop gentamicin	*Brucella* sp. rule out, in addition to serology testing
43	Intra-abdominal	Negative	Negative	Stop metronidazole, cefepime	Collection drained appeared stable by CT scan
49	Intrathoracic	Negative	*Klebsiella pneumoniae*, CoNS, *C. acnes*	Stop linezolid, minocycline	Coverage for *Nocardia* sp. lung nodules was removed
49	Bone, joint	Negative	*Cutibacterium acnes*	Stop ceftriaxone	Previously positive for *C. acnes* and CoNS
68	Bone, joint	Negative	Negative	Stop doxycycline	GNR on Gram stain; no sign of infection

^*a*^ CoNS, coagulase-negative staphylococci. CT, computed tomography. GPR, Gram-positive rod. GNR, Gram-negative rod. h/o, history of. I&D, incision and drainage. IVDU, intravenous drug use. MSSA, methicillin susceptible S. aureus. PJI, prosthetic joint infection. PVE, prosthetic valve endocarditis. SOT, solid organ transplant. s/p, status post.

^*b*^ Antimicrobial therapy change: (before) > (after) BR-PCR results. Antimicrobials: Amox-Clav, amoxicillin-clavulanic acid. Azithro, azithromycin. CTX, ceftriaxone. Dapto, daptomycin. Doxy, doxycycline. Erta, ertapenem. Levo, levofloxacin. Mero, meropenem. Mino, minocycline. Moxi, moxifloxacin. Posa, posaconazole. Vanc, vancomycin. Vori, voriconazole.

^*c*^ No appropriate microbiology cultures (fungal, AFB, anaerobic, or none) ordered to recover this organism.

## DISCUSSION

We hope that the advancements in molecular technologies translate into improved diagnostic accuracy, but usage guidelines for new molecular tests have lagged in part because their application, interpretation, and outcomes remain unclear (12). In this study, we evaluated the ability of BR-PCR results to influence changes in clinical management in more than 300 specimens from academic medical system hospitals over a 4-year period from 2018 to 2021. During this time, there was no oversight in the stewardship of BR-PCR testing. We found a change in clinical management in only 6% of the cases with BR-PCR results, as reflected by a change in antimicrobial therapy or duration (4.6%) or discontinuation of treatment (1.4%). This study shows a clinical impact in a similar proportion of BR-PCR results when compared to prior studies unfiltered for specimen type (4.1%, 44/1,062 samples [13]; 6.8%, 48/708 tests [16]; 6%, 15/247 samples [17]; 5%, 19/382 [18]).

Over half of the cases (11/21) prompting a change in clinical management identified bacteria from culture-negative specimens, representing bone and joint, CNS, intra-abdominal, and endovascular specimens. In 16/21 of these cases, an organism was detected by BR-PCR. Notably, 4 of these 16 specimens were not sent for routine culture, perhaps due to low suspicion for infection, failure to consult an ID team, or limited knowledge of test efficacy. Fungal and AFB BR-PCR results led to clinically meaningful changes in 3/21 cases. In 5/21 cases with a change in clinical management, cessation of antimicrobial therapy followed negative BR-PCR results.

Bacterial BR-PCR had a sensitivity of 30.9%; however, it was shown to complement the standard of care by addressing culture limitations to detect fastidious or uncommonly isolated microbes. PPA was 26%, which may reflect antimicrobial pretreatment and suggest a high rate of false positives. Routine reporting of contaminants and flora should be expected. Our data further supports published findings that BR-PCR is useful in identifying bacterial organisms from patients pre-treated with antibiotics and culture-negative specimens (15, 24, 25), and specimens with detectable inflammation and/or organisms are more likely to have positive bacterial identification by BR-PCR (13, 16, 18, 20).

Culture-negative specimens with positive BR-PCR were more often found in pre-treated patients than other discordant results. This suggests that culture-negative samples may have better yield for bacterial detection by BR-PCR if the patient had previous antibiotic exposure. This association may result from a higher likelihood of infection in patients who received antibiotic treatment prior to specimen collection (e.g., patients at higher risk for or who had clinical evidence of infection). Discordance in culture and BR-PCR results may also be impacted by sampling error. The microbiology lab often receives multiple samples taken from one infected body site, as is recommended (4, 12), but this is not routinely done for molecular testing. Thus, BR-PCR results should be carefully considered within the clinical scenario when attempting to rule out infection or stop therapy.

In some cases, BR-PCR elucidated a pathogen, but there was no change in clinical management. A lack of clinical utility was influenced by many factors, such as when (1) accurate and effective empiric therapy was already in place (2); a diagnosis was made by other methods (3); the patient was discharged, expired, or lost to follow-up; or (4) results were not considered clinically significant. These scenarios were at play for the following cases where BR-PCR detected: *Bartonella quintana* from endovascular tissue (trench fever; positive serology known 1 month prior to sending BR-PCR), *Coxiella burnetii* from endovascular tissue (Q fever; positive serology results available before BR-PCR results), *Streptobacillus moniliformis* from brain tissue (rat bite fever; isolated from positive blood culture 2 months prior), and *Brachyspira aalborgi* from intra-abdominal tissue (intestinal spirochetosis; spirochetes observed by pathology 5 months prior).

Constraints on the utility of BR-PCR include TATs approaching 3 weeks and inability to order it in routine clinical diagnostic laboratories. A potential limitation to the study may be the incorrect assumption that BR-PCR results prompted a change in management in cases where the availability of BR-PCR results coincided with the completion of a prescribed antibiotic therapy. Despite these limitations, the real-world use of BR-PCR in clinical care shows that this test can be meaningful, but it suggests that traditional infectious disease workup should be fully utilized before BR-PCR.

In current practice, we delay BR-PCR testing until bacterial culture is confirmed negative and fungal and AFB cultures have been reviewed, to prevent duplicate findings of organisms that grow relatively quickly. BR-PCR test ordering is limited to the ID specialty and must be approved by clinical microbiology directors. This framework supports the ordering of appropriate routine diagnostic tests and prevents unnecessary testing. We suspect that with oversight, the clinical utility of BR-PCR results will be greater, as studies focusing on BR-PCR results from culture-negative specimens have shown that a greater proportion of tests influences clinical utility (40.5%, 30/74 [19]; 25%, 18/71 [20]). At the current moment, BR-PCR should not replace the standard of care but provide a benefit for its ability to detect organisms from culture-negative specimens or fastidious/uncommonly isolated organisms. The low incidence in detection of fungi and AFB in clinical specimens by BR-PCR suggests a larger study is needed to determine the performance and clinical impact of these tests. Future case-control or multi-institutional studies design may better inform broadly applicable practices and guidelines for the use of BR-PCR in the diagnosis of infectious diseases and treatment of patients.

## Data Availability

This data set includes deidentified demographic, clinical test orders and results with date and times for calculation of TATs. Demographic, clinical and calculated TATs are available upon request.

## SUPPLEMENTAL MATERIAL

Supplemental material spectrum.02505-24-s0001.xlsx Tables S1 to S7.

ASM does not own the copyrights to Supplemental Material that may be linked to, or accessed through, an article. The authors have granted ASM a non-exclusive, world-wide license to publish the Supplemental Material files. Please contact the corresponding author directly for reuse.

## References

[1] Austin B. 2017. The value of cultures to modern microbiology. Antonie Van Leeuwenhoek 110:1247–1256. doi:10.1007/s10482-017-0840-8 28168566

[2] Lagier J-C, Edouard S, Pagnier I, Mediannikov O, Drancourt M, Raoult D. 2015. Current and past strategies for bacterial culture in clinical microbiology. Clin Microbiol Rev 28:208–236. doi:10.1128/CMR.00110-14 25567228 PMC4284306

[3] Scheer CS, Fuchs C, Gründling M, Vollmer M, Bast J, Bohnert JA, Zimmermann K, Hahnenkamp K, Rehberg S, Kuhn S-O. 2019. Impact of antibiotic administration on blood culture positivity at the beginning of sepsis: a prospective clinical cohort study. Clin Microbiol Infect 25:326–331. doi:10.1016/j.cmi.2018.05.016 29879482

[4] Osmon DR, Berbari EF, Berendt AR, Lew D, Zimmerli W, Steckelberg JM, Rao N, Hanssen A, Wilson WR, Infectious Diseases Society of America. 2013. Diagnosis and management of prosthetic joint infection: clinical practice guidelines by the Infectious Diseases Society of America. Clin Infect Dis 56:e1–e25. doi:10.1093/cid/cis803 23223583

[5] Baddour LM, Wilson WR, Bayer AS, Fowler VG Jr, Tleyjeh IM, Rybak MJ, Barsic B, Lockhart PB, Gewitz MH, Levison ME, Bolger AF, Steckelberg JM, Baltimore RS, Fink AM, O’Gara P, Taubert KA, on behalf of the American Heart Association Committee on Rheumatic Fever, Endocarditis, and Kawasaki Disease of the Council on Cardiovascular Disease in the Young, Council on Clinical Cardiology, Council on Cardiovascular Surgery and Anesthesia, and Stroke Council. 2015. Infective endocarditis in adults: diagnosis, antimicrobial therapy, and management of complications. Circulation 132:1435–1486. doi:10.1161/CIR.0000000000000296 26373316

[6] Rassoulian Barrett S, Hoffman NG, Rosenthal C, Bryan A, Marshall DA, Lieberman J, Greninger AL, Peddu V, Cookson BT, Salipante SJ. 2020. Sensitive identification of bacterial DNA in clinical specimens by broad-range 16S rRNA gene enrichment. J Clin Microbiol 58:e01605-20. doi:10.1128/JCM.01605-20 33028602 PMC7685877

[7] Salipante SJ, Sengupta DJ, Rosenthal C, Costa G, Spangler J, Sims EH, Jacobs MA, Miller SI, Hoogestraat DR, Cookson BT, McCoy C, Matsen FA, Shendure J, Lee CC, Harkins TT, Hoffman NG. 2013. Rapid 16S rRNA next-generation sequencing of polymicrobial clinical samples for diagnosis of complex bacterial infections. PLoS One 8:e65226. doi:10.1371/journal.pone.0065226 23734239 PMC3666980

[8] Chen YC, Eisner JD, Kattar MM, Rassoulian-Barrett SL, LaFe K, Yarfitz SL, Limaye AP, Cookson BT. 2000. Identification of medically important yeasts using PCR-based detection of DNA sequence polymorphisms in the internal transcribed spacer 2 region of the rRNA genes. J Clin Microbiol 38:2302–2310. doi:10.1128/JCM.38.6.2302-2310.2000 10834993 PMC86787

[9] Chen Y-C, Eisner JD, Kattar MM, Rassoulian-Barrett SL, Lafe K, Bui U, Limaye AP, Cookson BT. 2001. Polymorphic internal transcribed spacer region 1 DNA sequences identify medically important yeasts. J Clin Microbiol 39:4042–4051. doi:10.1128/JCM.39.11.4042-4051.2001 11682528 PMC88485

[10] Rakeman JL, Bui U, Lafe K, Chen Y-C, Honeycutt RJ, Cookson BT. 2005. Multilocus DNA sequence comparisons rapidly identify pathogenic molds. J Clin Microbiol 43:3324–3333. doi:10.1128/JCM.43.7.3324-3333.2005 16000456 PMC1169180

[11] Acid-fast bacilli (AFB) identification by sequence analysis. UW dept of lab medicine and pathology. 2022. Available from: https://depts.washington.edu/molmicdx/mdx/tests/afb.shtml

[12] Miller JM, Binnicker MJ, Campbell S, Carroll KC, Chapin KC, Gonzalez MD, Harrington A, Jerris RC, Kehl SC, Leal SM, Patel R, Pritt BS, Richter SS, Robinson-Dunn B, Snyder JW, Telford S, Theel ES, Thomson RB, Weinstein MP, Yao JD. 2024. Guide to utilization of the microbiology laboratory for diagnosis of infectious diseases: 2024 update by the infectious diseases society of America (IDSA) and the American society for microbiology (ASM). Clin Infect Dis:ciae104. doi:10.1093/cid/ciae104 38442248

[13] Kerkhoff AD, Rutishauser RL, Miller S, Babik JM. 2020. Clinical utility of universal broad-range polymerase chain reaction amplicon sequencing for pathogen identification: a retrospective cohort study. Clin Infect Dis 71:1554–1557. doi:10.1093/cid/ciz1245 31907545 PMC7486847

[14] Wang C-X, Huang Z, Fang X, Li W, Yang B, Zhang W. 2020. Comparison of broad-range polymerase chain reaction and metagenomic next-generation sequencing for the diagnosis of prosthetic joint infection. Int J Infect Dis 95:8–12. doi:10.1016/j.ijid.2020.03.055 32251799

[15] Rampini SK, Bloemberg GV, Keller PM, Büchler AC, Dollenmaier G, Speck RF, Böttger EC. 2011. Broad-range 16S rRNA gene polymerase chain reaction for diagnosis of culture-negative bacterial infections. Clin Infect Dis 53:1245–1251. doi:10.1093/cid/cir692 21976460

[16] Kubiak J, Morgan A, Kirmaier A, Arnaout R, Riedel S. 2023. Universal PCR for bacteria, mycobacteria, and fungi: a 10-year retrospective review of clinical indications and patient outcomes. J Clin Microbiol 61:e0095223. doi:10.1128/jcm.00952-23 38014970 PMC10729690

[17] Lucas EJ, Leber A, Ardura MI. 2019. Broad-range PCR application in a large academic pediatric center: clinical value and challenges in diagnosis of infectious diseases. Pediatr Infect Dis J 38:786–790. doi:10.1097/INF.0000000000002308 30920482

[18] Naureckas Li C, Nakamura MM. 2022. Utility of broad-range PCR sequencing for infectious diseases clinical decision making: a pediatric center experience. J Clin Microbiol 60:e0243721. doi:10.1128/jcm.02437-21 35400176 PMC9116169

[19] Lim PPC, Stempak LM, Malay S, Moore LN, Cherian SSS, Desai AP. 2022. Determining the clinical utility of 16S rRNA sequencing in the management of culture-negative pediatric infections. Antibiotics (Basel) 11:159. doi:10.3390/antibiotics11020159 35203762 PMC8868208

[20] Basein T, Gardiner BJ, Andujar Vazquez GM, Joel Chandranesan AS, Rabson AR, Doron S, Snydman DR. 2018. Microbial identification using DNA target amplification and sequencing: clinical utility and impact on patient management. Open Forum Infect Dis 5:ofy257. doi:10.1093/ofid/ofy257 30539032 PMC6284463

[21] Gay L, Melenotte C, Lakbar I, Mezouar S, Devaux C, Raoult D, Bendiane M-K, Leone M, Mège J-L. 2021. Sexual dimorphism and gender in infectious diseases. Front Immunol 12:698121. doi:10.3389/fimmu.2021.698121 34367158 PMC8339590

[22] Jenks JD, Aneke CI, Al-Obaidi MM, Egger M, Garcia L, Gaines T, Hoenigl M, Thompson GR 3rd. 2023. Race and ethnicity: risk factors for fungal infections? PLoS Pathog 19:e1011025. doi:10.1371/journal.ppat.1011025 36602962 PMC9815636

[23] Darwitz BP, Genito CJ, Thurlow LR. 2024. Triple threat: how diabetes results in worsened bacterial infections. Infect Immun 92:e0050923. doi:10.1128/iai.00509-23 38526063 PMC11385445

[24] Fournier P-E, Thuny F, Richet H, Lepidi H, Casalta J-P, Arzouni J-P, Maurin M, Célard M, Mainardi J-L, Caus T, Collart F, Habib G, Raoult D. 2010. Comprehensive diagnostic strategy for blood culture-negative endocarditis: a prospective study of 819 new cases. Clin Infect Dis 51:131–140. doi:10.1086/653675 20540619

[25] Voldstedlund M, Nørum Pedersen L, Baandrup U, Klaaborg KE, Fuursted K. 2008. Broad-range PCR and sequencing in routine diagnosis of infective endocarditis. APMIS 116:190–198. doi:10.1111/j.1600-0463.2008.00942.x 18377584

